# Microvascular mechanisms by which the combretastatin A-4 derivative AC7700 (AVE8062) induces tumour blood flow stasis

**DOI:** 10.1038/sj.bjc.6601261

**Published:** 2003-09-30

**Authors:** K Hori, S Saito

**Affiliations:** 1 Department of Vascular Biology, Division of Cancer Control, Institute of Development, Aging and Cancer, Tohoku University, 4-1 Seiryomachi, Sendai 980-8575, Japan

**Keywords:** combretastatin A-4, tumour blood flow, tumour vessel, interstitial fluid pressure, haemolysis

## Abstract

We previously reported that a novel combretastatin A-4 derivative, AC7700, has remarkable antitumour effects because of an irreversible stasis of tumour blood flow (TBF) and subsequent loss of nutrient supply to tumour tissue. Since early 2002, under the new designation AVE8062, AC7700 has undergone clinical trials in Europe and the US. Questions remain, however, concerning how AC7700 blocks TBF and why the TBF stasis does not recover. In this study, using a rat tumour LY80, a variant of Yoshida sarcoma, we examined whether TBF cessation after AC7700 administration is due to a direct action of the agent on tumour blood vessels. We constructed electrodes that can drop a small quantity of the drug solution directly at the site of blood flow measurement and inserted them subcutaneously and into the tumour. We compared the blood flow responses of normal vessels and tumour vessels after administration of 10-*μ*l doses of various concentrations (0.2, 1, 10, and 50 mg ml^−1^) of the AC7700 solution. In addition, we assessed TBF stasis after i.v. and intra-arterial 10 mg kg^−1^ AC7700 administration in an LY80-induced kidney tumour. To determine why the TBF stasis is irreversible, we observed AC7700-induced changes in host arterioles and the tumour vascular network of the Sato lung carcinoma using a vital microscopic rat transparent chamber. Since an increase in tumour interstitial fluid pressure brings about a decrease in TBF, we also measured 10 mg kg^−1^ AC7700-induced changes in this pressure. The sensitivity of the blood flow response after intratumoral application of AC7700 was markedly higher in normal vessels relative to tumour vessels. Intra-arterial administration of AC7700 did not have stronger effects on TBF stasis than did i.v. administration. Intravital microscopy showed that AC7700 induced a powerful and long-lasting constriction of host arterioles, so that complete stasis of blood flow occurred in downstream vessels, which supplied blood to tumours. Owing to this stasis, the lumens of numerous tumour vessels narrowed or completely disappeared, and numerous erythrocytes stagnated in drainage vessels of the tumour vascular network. Haemolysis of these erythrocytes occurred after 2–3 h, resulting in complete thrombosis. There was no indication of reperfusion in vessels showing haemolysis. This haemolysis is thought to be the main cause for the irreversibility of TBF stasis. Since the tumour interstitial fluid pressure decreased after i.v. AC7700 administration, the possibility of stasis of TBF being caused by tumour vascular compression was excluded. All these results strongly suggest that the main target of AC7700 is host arterioles and that the stasis of TBF induced by AC7700 is not triggered by a direct action of the drug on tumour vessels.

Combretastatin A-4 (CA-4) is an antimitotic agent with *in vitro* inhibitory action on tubulin polymerisation. It was first isolated from the bark of the South African bush willow by Pettit *et al* (1989), after which the water-soluble prodrug combretastatin A-4 phosphate (CA-4-P) was synthesised (Pettit *et al*, 1995), and the effects of CA-4 were studied *in vivo* (Dark *et al*, 1997). Combretastatin A-4 was determined to have strong suppressive effects on tumour blood flow (TBF) (Tozer *et al*, 1999), and its ability to induce widespread necrosis of solid tumours was described in several studies (Dark *et al*, 1997; Beauregard *et al*, 1998; Horsman *et al*, 1998; Tozer *et al*, 1999; Zhao *et al*, 1999; Landuyt *et al*, 2000; Malcontenti-Wilson *et al*, 2001). Recently, Tozer *et al* (2001, 2002) reported the mechanism of action of CA-4-P, which is currently undergoing phase I and phase II clinical trials (Dowlati *et al*, 2002).

As the structure of CA-4 is simple, many derivatives have been synthesised since the discovery of this substance by Pettit *et al* (1989). In recent years, AC7700, a combretastatin analogue, was developed in Japan (Hatanaka *et al*, 1998; Ohsumi *et al*, 1998) and was found to have more powerful TBF stasis effects (Hori *et al*, unpublished data) and antitumour effects (Nihei *et al*, 1999b) compared with CA-4-P. Using subcutaneously (s.c.) transplanted tumours in rodents and histological methods, we previously showed that not only is AC7700 effective and suppresses tumour proliferation (Hori *et al*, 1999; Nihei *et al*, 1999a) but it also significantly prolongs survival in tumour-bearing rats (Hori *et al*, 1999). AC7700 works both in rapidly proliferating transplanted tumours and in relatively slowly proliferating primary tumours induced by chemical carcinogens (Hori *et al*, 2001). Recently, such effectiveness was confirmed in tumours growing within internal organs, including the liver, kidney, and stomach, as well as in metastatic lymph nodes and small 2.5-mm-diameter foci (Hori *et al*, 2002), which suggests that these effects might be obtained in all types of solid tumours. Since early 2002, AC7700 has been undergoing clinical trials in Europe and the US under the new code name AVE8062.

It is clear that the strong antitumour effects of AC7700 are due to blocking of the nutritional supply to the tumour by means of TBF stasis, but many questions remain about the exact mechanism of the stasis. The stasis may be brought about by a direct action of AC7700 on the tumour vessels. Alternatively, the stasis may occur indirectly via the host's vascular response. However, *in vivo* demonstration of this effect has not been achieved. Moreover, it remains uncertain why TBF does not easily recover after the stasis.

The purposes of the present study were four-fold: (a) to determine whether the cause of TBF stasis induced by AC7700 is the result of a direct action of this agent on tumour vessels; (b) to determine by means of intravital microscopy why the drug-induced stasis is irreversible; (c) to determine whether AC7700, like CA-4-P, can increase tumour vessel permeability and thereby increase tumour interstitial fluid pressure (TIFP); and (d) to construct a self-consistent model of the various phenomena observed after AC7700 administration and thereby elucidate the microcirculatory mechanisms that lead to irreversible TBF stasis and subsequent necrosis.

## MATERIALS AND METHODS

### Rats and tumours

Male Donryu rats (Crj-Donryu; Nippon Charles-River, Yokohama, Japan), 8–10 weeks old and with an average weight of 250–300 g, were used for all experiments. Rats were bred and maintained in a ventilated, temperature-controlled (24±1°C), specific pathogen-free environment on a bed of wood shavings with food and water freely available and a 12-h light–dark cycle. They were usually housed two or three per cage. The rats equipped with transparent chambers for microscopic observations (see below) and the rats fitted with diffusion chambers for measurements of TIFP (see below) were caged singly.

Tumour cell lines included LY80, which is a variant of the Yoshida sarcoma, and Sato lung carcinoma (SLC), which is an undifferentiated lung carcinoma. In our laboratory, LY80 and SLC are maintained by successive i.p. and s.c. transplantations, respectively. Although SLC and LY80 showed almost the same growth potential in the transparent chamber and showed almost the same reaction after AC7700 administration, we chose SLC rather than LY80 for vital microscopic observations. We did so because when SLC is growing in transparent chambers, demarcation between the edge of the growing tumour and the normal tissue is clear (Hori *et al*, 1995), and therefore the change in normal arterioles and tumour vessels after AC7700 administration can easily be recognised.

All experimental protocols were reviewed by the Committee on the Ethics of Animal Experiments in our institute and were carried out in accordance with Guidelines for Animal Experiments issued by Tohoku University School of Medicine and The Law (No. 105) and Notification (No. 6) issued by the Japanese Government. The ethical guidelines that were followed meet the standards required by the UKCCCR (Workman *et al*, 1998) guidelines.

### Tumour cell implantation

LY80 cells growing in ascites of a donor rat were collected, suspended in pH 7.4 phosphate-buffered saline, and adjusted to a concentration of 2 × 10^6^ cells per 0.1 ml or 10 *μ*l. Recipient rats were anaesthetised with diethyl ether (Wako Pure Chemical Industries, Ltd, Osaka, Japan). For the investigation of topical application of the drug to the tumour tissue, and for the experiment measuring TIFP using the wick-in-needle method, the tumour cell suspension was injected s.c. into the back of each rat. For measuring TIFP by the diffusion chamber method, the chamber was first implanted in the back of the rat and then a suspension (0.1 ml) containing 2 × 10^6^ cells was injected around the chamber. For the experiment investigating the effect of intra-arterial (i.a.) AC7700 injection, tumour cells were implanted into the kidney. Recipient rats were anaesthetised and placed in the left lateral decubitus position, and a vertical incision was made in the right flank through the skin and peritoneum to expose the lateral aspect of the kidney. The tumour cells (2 × 10^6^ cells in 10 *μ*l) were implanted into the renal parenchyma using a 50-*μ*l graduated syringe (Hamilton Co., Reno, NV, USA) and a 27-gauge syringe needle (27 G; Termo Co., Tokyo, Japan). The site of injection was 2 mm below the renal capsule. The hole made by the needle was sealed with synthetic resin glue (Aron Alpha 201; Toagosei Chemical Industry Co., Tokyo, Japan). After the injection, the incision wound was closed in one layer with thread, and the animals were allowed to recover.

### Drugs

AC7700 (AVE8062), one of the combretastatin derivatives, was synthesised and provided by Ajinomoto Pharmaceutical Research Laboratories, Kawasaki, Japan. The AC7700 powder was dissolved in 0.9% NaCl solution immediately before use. The AC7700 solution was usually injected into the tail vein at a rate of 0.15 ml min^−1^ using an infusion pump (Compact Syringe Pump; Harvard Apparatus Co., Inc., Millis, MA, USA). In all experiments of the present study, the AC7700 administration was completed within 2 min. The solution volume administered was 1 ml kg^−1^. Fluorescein isothiocyanate dextran (molecular weight (m.w.) 4400 Da) (FITC-dextran, Sigma Chemical Co., St Louis, MO, USA) was used as a fluorescent contrast material. Experiments were performed with the animals anaesthetised in a temperature-controlled (24±1°C) box fitted with a suction duct. Both pentobarbital sodium salt (Tokyo Kasei Kogyo Co., Ltd, Tokyo, Japan) and enflurane (Ethrane; Abbott Laboratories, North Chicago, IL, USA) were used simultaneously for anaesthesia. The pentobarbital powder was solved in distilled water (Otsuka Pharmaceutical Co., Ltd, Tokyo, Japan) to give a concentration of 50 mg ml^−1^ and was administered intramuscularly (i.m.) at a dose of 30 mg kg^−1^ 10 min before the experiment. When the experiment continued for more than 2 h, supplemental doses (15 mg kg^−1^ i.m.) were given at 90-min intervals to maintain immobilisation. Enflurane concentration was maintained at 1% in the inhaled carrier gas, which was administered at a rate of 1 l min^−1^ by means of an anaesthetic apparatus for small laboratory animals (Hori *et al*, 1991). We certify that this anaesthetic condition did not change blood pressure and TBF significantly throughout the experiment of 6 h (Hori *et al*, 2002).

### Measurement of TBF

The tumour blood flow was measured using the hydrogen clearance method (Hori *et al*, 1991, 1996, 2002) that was originally developed by Aukland *et al* (1964). The blood flow measured by this method is not the total blood flow (Gullino and Grantham, 1961); rather, it is the local blood flow (Peterson, 1979). In brief, after saturation of the tissue with hydrogen following inhalation of 9% hydrogen gas in air (at 1 l min^−1^), the blood flow value (in ml min^−1^ (100 g tissue)^−1^) in a small region was calculated from the half-life of the clearance curve obtained. A tissue blood flow meter with two separate amplifiers (PHG-201; Unique Medical Co., Tokyo, Japan) was used. Two 80-*μ*m-diameter hydrogen electrodes (UHE-201C; Unique Medical) and two rod-type Ag/AgCl reference electrodes (TT-98012; Unique Medical) were usually used for each rat. The reference electrodes were inserted between the skin and musculature in the caudal region. In the experiment measuring TIFP, one electrode was used to monitor TBF. In the experiment measuring TBF change after topical application of AC7700, a special electrode (see below) was used.

The tumour blood flow was measured 7–10 days after tumour implantation. For measuring TBF in the kidney tumour, a vertical incision was carefully made in the right flank through the skin and peritoneum, similar to the incision for implantation of the tumour, and electrodes were inserted into the solid tumour growing in the renal parenchyma, to a depth of 2 mm from the tumour surface. Throughout the experiment, rats were placed prone on a heated stage at 34°C and kept in the same position. The rectal temperature monitored with a thermistor for small animals (PTC-201; Unique Medical) was 33.5–35.5°C and the condition was maintained.

### Measurement of mean arterial blood pressure

The mean arterial blood pressure (MABP) was monitored in all rats in which TBF was measured. The mean arterial blood pressure was measured via a catheter (PE-50; Clay Adams, Persippany, NJ, USA) inserted into the right femoral artery. Pressure in the catheter was recorded with a pressure transducer (TNF-R; Spectramed Medical Products, Singapore), the output of which was fed into an amplifier (6M82; NEC-Sanei Co., Tokyo, Japan) adapted for such a measurement.

### Development of a novel electrode for measuring changes in local blood flow after topical application of AC7700

To investigate whether tumour vessels or normal vessels are more sensitive to AC7700, we constructed a new type of wire electrode for use in the hydrogen clearance method. From the tip of the wire, a section 1–10 mm long is enveloped by a thin polyethylene tube (outer diameter, 180 *μ*m). The sensitive part of the electrode is the 1-mm tip, which detects hydrogen gas. At 10 mm from the tip, the wire is passed through a pinhole made in the sidewall of the tube. The pinhole is packed with silicone rubber. With this electrode, we were able to measure the dose–response relationship for AC7700.

### Topical application of AC7700 and 0.9% NaCl solution into tumours and normal s.c. tissue

For tumours, the tip of the electrode was placed at a depth of 4 mm below the tumour surface. For normal tissue, the electrode was introduced subcutaneously into the animal's back via a syringe needle (18 G, Termo Co.). The tail end of the polyethylene tube was connected to a 50-*μ*l graduated syringe (Hamilton Co.). Through this tube, 10 *μ*l of AC7700 solution (0.2, 1, 10, or 50 mg ml^−1^) or 10 *μ*l of 0.9% NaCl solution was dropped directly on the region at which blood flow would be measured.

Before the topical application of each solution, blood flow was measured twice at 30-min intervals. When the blood flow had stabilised, the solution was dropped on the region of measurement, and 30 min later the blood flow was measured again. The rate of change of blood flow (%) was calculated from the values before and 30 min after the topical application of the solution.

For direct observation of the contraction of normal arterioles caused by AC7700, intravital microscopy was used to drop 10 *μ*l of 0.2 mg ml^−1^ AC7700 solution on the vessels of the s.c. tissue, and the measurements were recorded.

### Comparison of changes in TBF induced by i.a. and i.v. administration of AC7700

For this comparison, LY80 cells (2 × 10^6^) were implanted in the right kidney as described earlier. At 7–10 days after the implantation, the second incision was made at exactly the same incision site as that of the first incision, the kidney was exposed, and hydrogen electrodes were inserted into the tumour growing there. Next, a polyethylene tube with an outer diameter of 0.61 mm (PE10; Clay Adams) was inserted into the left common carotid artery until the tip of the tube reached a position 2–3 mm upstream of the right renal artery. All incision wounds were sutured with thread or were closed with synthetic resin glue (Aron Alpha 201). Certain animals in the i.v. injection group also underwent this operation as a sham operation.

In both the i.a. injection group (*n*=6) and the i.v. injection group (*n*=6), 10 mg ml^−1^ AC7700 was infused at a rate of 0.15 ml min^−1^ using a microinfusion pump (Harvard Apparatus Co.). The final dose administered was 10 mg kg^−1^. After administration of AC7700, TBF was measured at multiple time points (i.e. 10, 30, and 60 min and every subsequent 1 h) for 6 h. In another experimental group, AC7700 was administered i.a. to rats at a dose of 0.5 and 1 mg kg^−1^.

### Transparent chamber assays

For direct observation of changes in normal arterioles and tumour vessels caused by AC7700, transparent chambers (Hori *et al*, 1990) were implanted in dorsal skin flaps of rats under aseptic conditions. The chamber used in the present experiment was a ‘sandwich’ system (Yamaura and Sato, 1974) in which each chamber consists of a pair of identical titanium frames containing a circular quartz glass window 300 *μ*m thick. The method of implantation of the chamber has been previously described in detail (Hori *et al*, 1981, 1990).

### Method of assignment of orders to arterioles

In the s.c. tissue of the rat, arterioles, terminal arterioles, and certain capillaries could be ordered according to Strahler's (1957) nomenclature, which is a purely topological method and has been used extensively in various applications, such as for rivers, trees, and blood vessels. Strahler's method is as follows: when two segments of the same order join, the parent segment is assigned the next higher order. If two daughter segments that have different orders are joined, the parent segment retains the higher of the two orders. In our experiments, capillaries originally branching off from terminal arterioles classified by Wiedeman (1984) were assigned order 1. Once those capillaries were defined as order 1, terminal arterioles were assigned order 2 (Hori *et al*, 1990). According to this nomenclature, the orders of arterioles were named a2, a3, a4, and so on, as one moves stepwise from the terminal arteriole upward.

### Vessel classification within a tumour vascular network

Tumour vessels within a transparent chamber were classified into one of the following three groups on the basis of their anatomical position and function: (a) feeding arterioles, which supply blood to a tumour vascular network; (b) tumour capillaries, which constitute most of a tumour vascular network and play an important role in nutrient exchange; and (c) drainage vessels, which drain blood from a tumour vascular network. We previously reported that TBF is controlled by modified a2 vessels (Hori *et al*, 1990). That is, feeding vessels are a2 arterioles modified by the tumour. The diameter of the vessels becomes wider compared to a2 arterioles. We observed in preliminary experiments that these three vessel groups can be differentiated according to their reactions to AC7700.

### Intravital microscopic observation of changes in tumour vessels and TBF caused by AC7700

After anaesthesia, a rat with a transparent chamber was placed in the right lateral position on a heated stage (MATS-SFA; Tokai HIT Co., Ltd, Tokyo, Japan) at 34.5°C, which was attached to the mechanical stage of the microscope. AC7700 (10 mg kg^−1^) was administered via the lateral tail vein using an infusion pump (Harvard Apparatus Co.). The changes in TBF and the tumour vascular network were directly observed via a light microscope (Eclipse E800; Nikon Corp., Tokyo, Japan) with a × 10 ocular (CFI UW; Nikon) and × 2-20 objectives (CFI Plan Fluor; Nikon Corp.). Tumour vessels within the chamber were transilluminated by a 12-V 100-W halogen lamp. The microscopic image was recorded using a closed-circuit video system consisting of a CCD video camera (CS-900; Olympus Kogaku K.K., Tokyo, Japan), a TV monitor (PVM-14M4J; Sony Corp., Tokyo, Japan), and an S-VHS video recorder (SVO-2100; Sony).

For intravital fluorescence microscopic observation, the light source was changed to a 100-W mercury lamp. A solution of 2% FITC-dextran (m.w. 4400 Da) was injected into the rat as a single i.v. bolus. Tumour vessels within the chamber were epi-illuminated through a primary filter (420–490 nm), a 505-nm dichroic interference mirror, and a 520-nm barrier filter. The fluorescein microscopic images were photographed using a silicone-intensified video camera (C2400-08, Hamamatsu Photonics, Hamamatsu, Japan).

A video timer was superimposed on the images for record keeping. Segments of the videotape containing desired images were transferred to the hard disk of a computer (Power Macintosh 8600/200, Apple Japan, Inc., Tokyo, Japan) through a video frame grabber board (IQ-V50PCI, Hamamatsu Photonics). Final images were output by a digital printer (Pictography 4000, Fuji Photo Film Co., Ltd, Tokyo, Japan).

### Measurement of arteriole and drainage diameters

Intravital microscopic observation for diameter measurements was performed under × 200 magnification. Changes in arteriole and drainage diameters caused by AC7700 were measured directly on the TV monitor. The rate of change in the diameter before and after the administration of AC7700 was calculated.

### Histological investigation of changes in tumour capillaries caused by AC7700

To confirm the presence of functioning tumour vessels, 2% FITC-dextran was injected i.v. with the use of intravital microscopy. When FITC-dextran disappeared from the tumour tissue 120 min later, 10 mg kg^−1^ AC7700 was administered. FITC-dextran was then injected 30 min after the AC7700 administration to demonstrate complete loss of circulatory function in the tumour vascular network. After an additional 90 min, the glass window of one side of a transparent chamber was gently removed from the rat, 15% formalin solution was put directly on the tissue (approximately 150 *μ*m thick), and the thin tissue was fixed before killing. The tissue was processed and embedded in paraffin. Sections (4 *μ*m thick) were cut in parallel to the surface of the membrane tissue within the transparent chamber and were stained with haematoxylin and eosin. Changes in microvessels caused by AC7700, in particular, changes in tumour vascular lumens, were carefully assessed.

### Changes in TIFP caused by AC7700

The tumour interstitial fluid pressure was measured by two different methods – the diffusion chamber method and the wick-in-needle method. Systemic arterial pressure and TBF were also simultaneously measured in both methods.

#### Diffusion chamber method.

We previously reported this method in detail (Hori *et al*, 1986). The apparatus fitted to the subcutis is composed of a diffusion chamber with a pore size of 0.45 *μ*m, developed originally by Gullino *et al* (1964), and a pair of perforated aluminium shields for fixing its position in the subcutis. The chamber was inserted into the s.c. tissue of the caudal portion of the skin flap. After insertion of the chamber, a suspension (0.3 ml) containing 2 × 10^6^ LY80 tumour cells was injected s.c. around it. The chamber was completely enveloped in the LY80 tumour approximately 1 week after transplantation of tumour cells. The TIFP was recorded electrically with a transducer (Spectramed Medical Products) through the polyethylene tube connected to the diffusion chamber. The tumour interstitial fluid pressure was serially measured and recorded before and after AC7700 administration.

#### Wick-in-needle method.

Measurement of TIFP by the wick-in-needle method was performed using the technique reported by Scholander *et al* (1968). In the present study, we used the wick-in-needle device made from a 23-gauge syringe needle (23 G, Termo Co.). The needle was inserted into the solid tumour, and the other end of the device was connected to a pressure transducer (Spectramed Medical Products) by a polyethylene tube. The tumour interstitial fluid pressure was serially measured and recorded before and after the administration of AC7700 as in the diffusion chamber method.

### Statistics

All results are expressed as means±s.d. The statistical significance of the difference in the blood flow reduction rate caused by topical application of each concentration of the AC7700 solution between tumours and normal s.c. tissue, and among the tumours or normal s.c. tissues, was evaluated with an unpaired two-group *t*-test. The significance of the difference in TBF reduction at each time point between i.v. and i.a. administration of AC7700 was evaluated with repeated measures ANOVA. Comparisons of the difference of diameter of arteriolar vessels before and after AC7700 administration were made by using paired two-group *t*-tests. *P*-values of 0.05 or lower were considered significant.

## RESULTS

### Topical application of AC7700 or 0.9% NaCl solution to tumours and normal s.c. tissues

Blood flow values measured by the special electrodes developed for topical application were not significantly different from those measured by electrodes that have been used so far (data not shown), which indicated that the slight increase in the diameter of the electrodes because of the covering wire did not greatly influence the blood flow measurement. When 10 *μ*l of the control (0.9% NaCl) solution was dropped directly at the site of blood flow measurement, the flow signal increased in a transient manner as an artefact; however, the blood flow returned to its original value within 5 min. Each blood flow response caused by topical application of each concentration of AC7700 was reproducible.

Figure 1A

**Figure 1:**
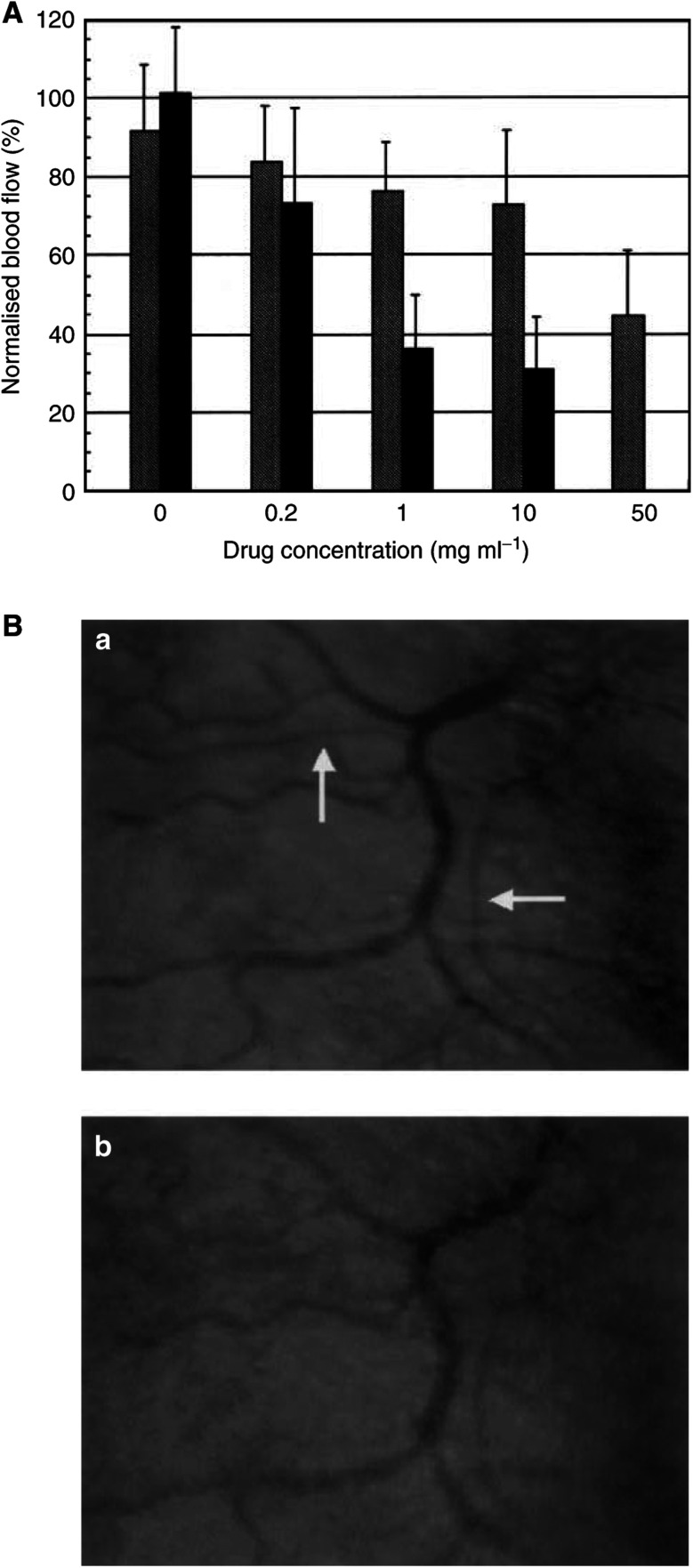
Changes in blood flow induced by topical application of AC7700 or 0.9% NaCl solution to tumours and normal s.c. tissues (**A**) A sample of 10 *μ*l of AC7700 solution at each concentration (0.2, 1, 10, and 50 mg ml^−1^) and of 0.9% NaCl solution was dropped on the region of blood flow measurement in tumour and s.c. tissue.The blood flow was measured before and 30 min after application of the solution, and the rate of change in blood flow (%) was calculated. Black bars, normal s.c. tissue; hatched bars, tumour. For each concentration of AC7700 and 0.9% NaCl solution, nine and seven rats were used for measurements in normal s.c. tissues and tumours, respectively. At concentrations of 1 and 10 mg ml^−1^, the sensitivity of normal s.c. tissue to AC7700 was significantly higher than that of tumour tissue. (**B**) vital microscopic images of changes in arterioles after topical application of 10 *μ*l of 0.2 mg ml^−1^ AC7700. Note that an arteriole (arrows) was markedly constricted by AC7700: (a) before droplet application; (b) 19 s after droplet application.

shows changes in local blood flow when 10 *μ*l of various concentrations (0.2, 1, 10, or 50 mg ml^−1^) of the AC7700 solution or 10 *μ*l of 0.9% NaCl solution was dropped directly at the site of blood flow measurement in normal s.c. tissues and s.c. tumours. Local blood flow of the normal s.c. tissues decreased significantly and dose dependently because of AC7700 (0.9% NaCl *vs* 0.2 mg ml^−1^ AC7700, *P*=0.0116; 0.9% NaCl *vs* 1 mg ml^−1^ AC7700, *P*<0.0001; 0.9% NaCl *vs* 10 mg ml^−1^ AC7700, *P*<0.0001). In contrast, local blood flow of LY80 tumours did not change significantly at any concentration of AC7700 (0.9% NaCl *vs* 0.2 mg ml^−1^ AC7700, *P*=0.3445; 0.9% NaCl *vs* 1 mg ml^−1^ AC7700, *P*=0.0706; 0.9% NaCl *vs* 10 mg ml^−1^ AC7700, *P*=0.0690). The TBF did decrease significantly with 50 mg ml^−1^ AC7700 (*P*=0.0002).

The concentration of AC7700 needed to reduce blood flow to one-half of the original value was 50 mg ml^−1^ in tumour tissue, whereas only 1 mg ml^−1^ was sufficient in normal tissue. That is, the sensitivity of the blood flow response after topical application of AC7700 was markedly higher in normal vessels relative to the tumour vessels. Even at the concentration of 0.2 mg ml^−1^, when AC7700 was applied directly to normal s.c. tissue, arterioles first constricted (Figure 1B) and the local blood flow decreased by approximately 30% (Figure 1A). In contrast, when the same concentration and volume of AC7700 were used for tumour tissue, TBF did not show significant changes (Figure 1A).

### Comparison of the changes in TBF induced by i.a. and i.v. administration of AC7700

**Figure 2:**
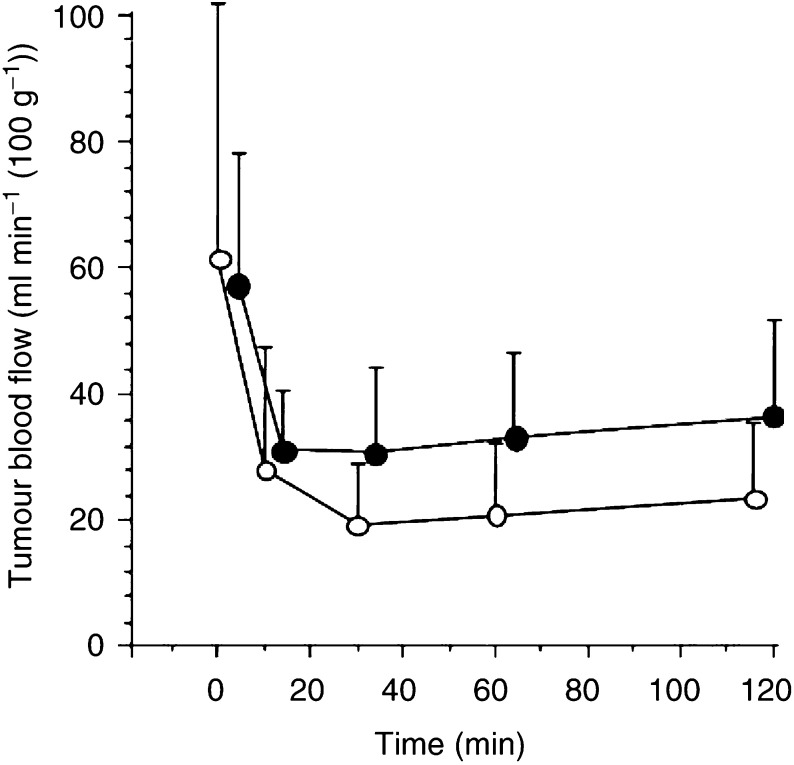
Comparison of changes in TBF after i.a. and i.v. administration of AC7700. AC7700 administration (10 mg kg^−1^) was completed at 0 min. The difference in TBF reduction between the two routes of AC7700 administration was not significant (*P*=0.0765): •, i.a. route (*n*=12); ○, i.v. route (*n*=11).

A comparison of changes in TBF after i.v. and i.a. administration of 10 mg kg^−1^ AC7700 is shown in Figure 2

. There was no significant difference between the two groups (*P*=0.0765). Intra-arterial administration of AC7700 did not have stronger effects than i.v. administration. For a dose of 1 mg kg^−1^ AC7700 or lower, i.a. administration did not induce a prominent decrease in TBF (data not shown).

### Changes in the diameter of host arterioles and tumour-feeding vessels caused by i.v. administration of AC7700

Table 1

**Table 1:** Effects of AC7700 on vessel diameter in each vascular segment in s.c. tissue

		**Vessel diameter (*μ*m)**		
**Vessel type**	***n***	**Before AC7700**	**30 min after AC7700**	**Change (%)**
a4	10	50.7±10.6	35.9±8.3^b^	−29.2
a3	18	30.7±8.3	20.4±6.3^b^	−33.6
a2	7	21.4±4.5	11.3±2.5^b^	−47.2
Feeding vessel	6	37.2±4.8	—c	

a Vessel diameter was measured before and 30 min after administration of 10 mg kg^−1^ AC7700. Values are expressed as means±s.d. Eight rats were used. *n*=number of measurements.

b *P*<0.0001.

c Impossible to measure.

shows changes in the diameter of arteriolar vessels and tumour-feeding vessels caused by AC7700. When 10 mg kg^−1^ AC7700 was given i.v., all orders of arterioles within a transparent chamber showed significant vasoconstriction (a4, a3, and a2, *P*<0.0001). The changes in diameters of a4, a3, and a2 vessels 30 min after AC7700 administration were −29.2, −33.6, and −47.2%, respectively. The blood flow in tumour-feeding vessels (modified a2 arterioles) ceased completely, and the vessels disappeared from view. Thus, it was impossible to measure the diameter of these vessels after AC7700 administration. The blood flow in normal tissue did not stop.

### Process of irreversible stasis of TBF caused by AC7700

**Figure 3:**
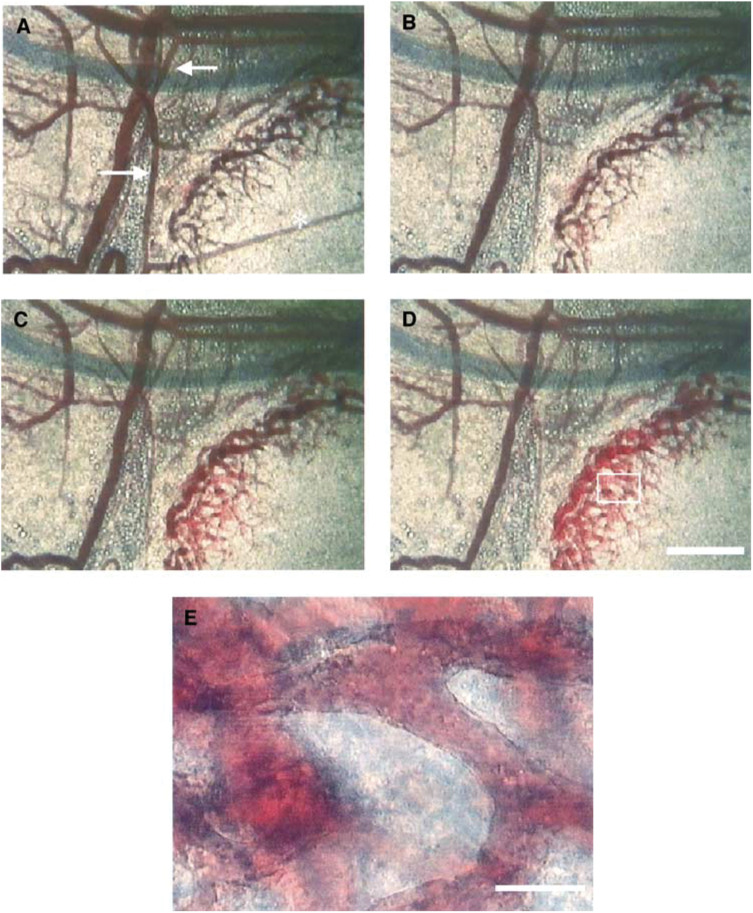
Process of irreversible TBF stasis caused by AC7700: (**A**) before 10 mg kg^−1^ AC7700 administration; (**B**) 30 min after the end of drug administration; (**C**) 2 h later; (**D**) 2.5 h later. (**E**) Enlargement of a section of **D**. (**A–D**) Photograph with the × 10 the eyepiece and the × 4 objective (bar, 500 *μ*m); (**E**) photograph with the × 10 the eyepiece and the × 20 the objective (bar, 100 *μ*m). Arterioles (arrows) and a feeding arteriole (^*^) showed marked contraction induced by AC7700. Tumour blood flow stopped completely 30 min after i.v. AC7700 administration, and many erythrocytes were trapped in drainage vessels located at the tumour periphery. Dramatic haemolysis occurred in those tumour vessels 2–2.5 h after drug administration (**C, D**). Haemorrhage was not observed (**E**). Reperfusion of blood into these tumour vessels was never seen.

A typical intravital microscopic finding showing the process of change in tumour microcirculation caused by AC7700 is shown in Figure 3

. When AC7700 was administered i.v., a3 and a4 arterioles near the tumour constricted (Figure 3B), and then MABP increased by 38%. The blood flow in tumour-feeding vessels (asterisk in Figure 3A) began to decrease immediately after AC7700 administration and stopped completely 30 min later. As a result, the TBF in the numerous tumour capillaries that formed the tumour vascular network ceased completely.

**Figure 4:**
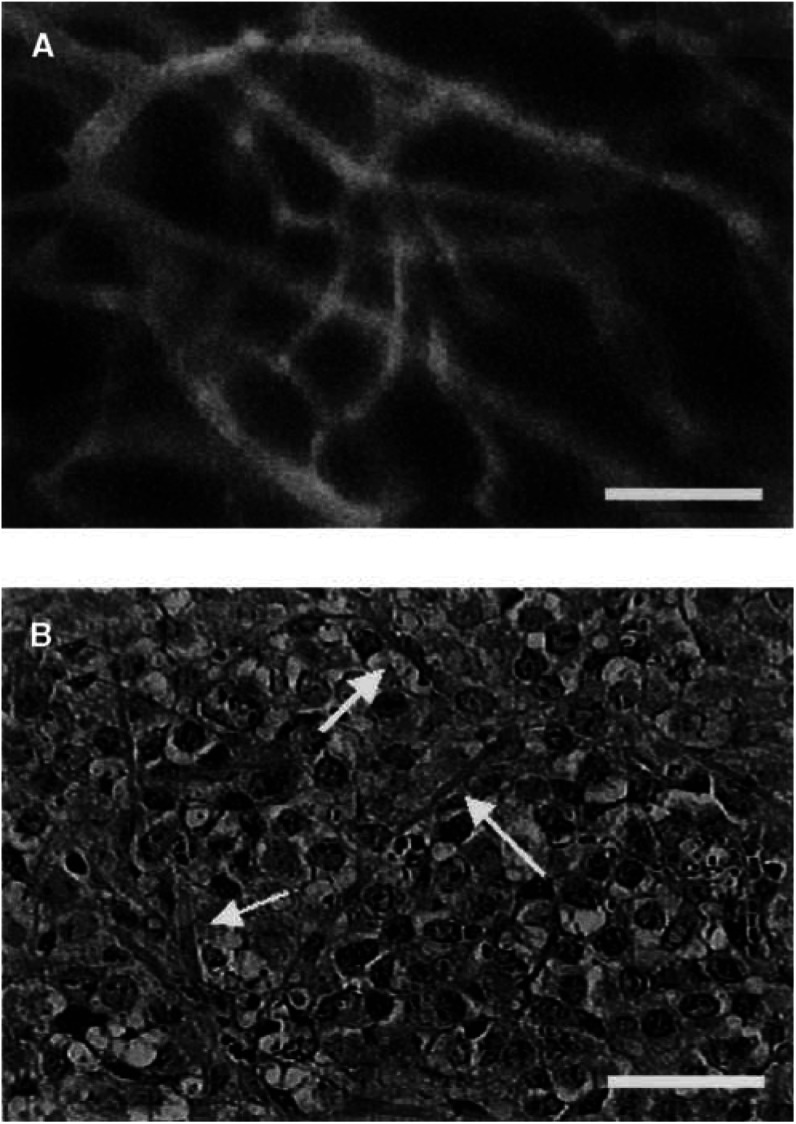
Disappearance of tumour vascular lumens caused by AC7700-induced TBF stasis. (**A**) Fluorescence angiography. To confirm functioning tumour vessels, 2% FITC-dextran (m.w. 4400 Da) was administered by a single bolus i.v. injection. Before AC7700 administration, the fluorescent dye did reach the tumour vessels. Many tumour vessels had diameters of 50–100 *μ*m. Bar, 250 *μ*m. (**B**) histology of the same area 120 min after administration of 10 mg kg^−1^ AC7700. The tumour tissue within the chamber was locally fixed by formalin before killing. Many tumour vessels greatly constricted or the vascular lumens disappeared, and the vessels showed a fine thread-like appearance. We confirmed this finding by serial section of the tissue. Bar, 50 *μ*m.

When FITC-dextran was administered by a single bolus i.v. injection before AC7700 administration, the fluorescent dye reached the tumour vessels (Figure 4A

). Many tumour vessels had diameters of 50–100 *μ*m. However, 30 min after AC7700 administration FITC-dextran did not reach the tumour vessels at all (data not shown) because of complete blockage of the tumour microcirculation. A histological section of the same area of the tumour tissue, which was locally fixed by 15% formalin before killing, is shown in Figure 4B. It should be noted that the vascular lumens of the tumour vessels narrowed or disappeared after AC7700-induced TBF stasis, and many vessels had a fine, thread-like appearance (Figure 4B, arrows).

**Figure 5:**
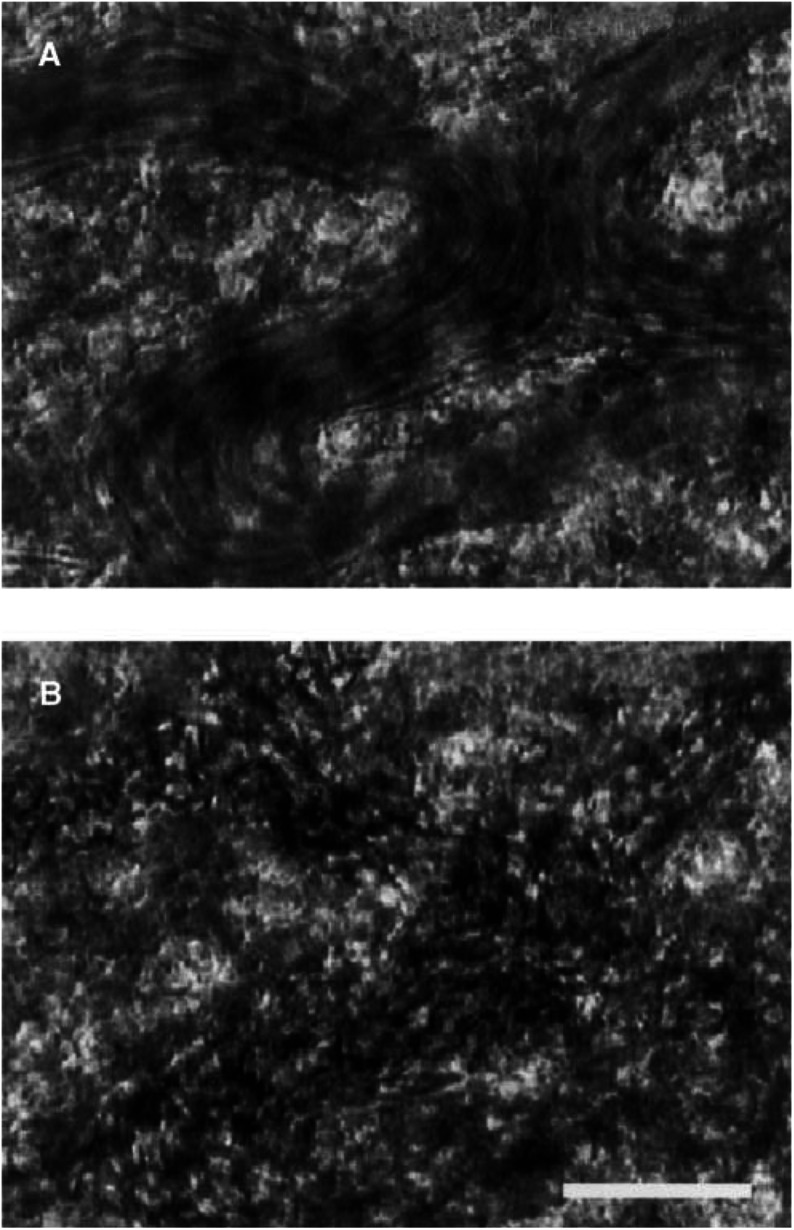
Stagnation of blood cells in the drainage vessels caused by AC7700: (**A**) before administration of 10 mg kg^−1^ AC7700; (**B**) 30 min after AC7700 administration. The plasma volume in tumour vessels gradually decreased, and many erythrocytes remained in the vessels. Bar, 100 *μ*m.

The drainage of the tumour vascular bed, which was located at the periphery of the tumour and frequently had a sinusoid-like structure, often showed a gradual decrease in plasma volume, and many erythrocytes remained there (Figure 5

). After 2–3 h, erythrocytes left in these drainage vessels underwent haemolysis (Figure 3C and D), resulting in passive enlargement of vascular diameter and thrombosis in the vessel lumens (Figure 3E). Once haemolysis-induced thrombosis occurred, the TBF never recovered. These phenomena were observed in all 12 tumours viewed in the transparent chambers, without exception. The change in the diameter of drainage vessels was +31.0±11.4% (11 chambers).

**Figure 6:**
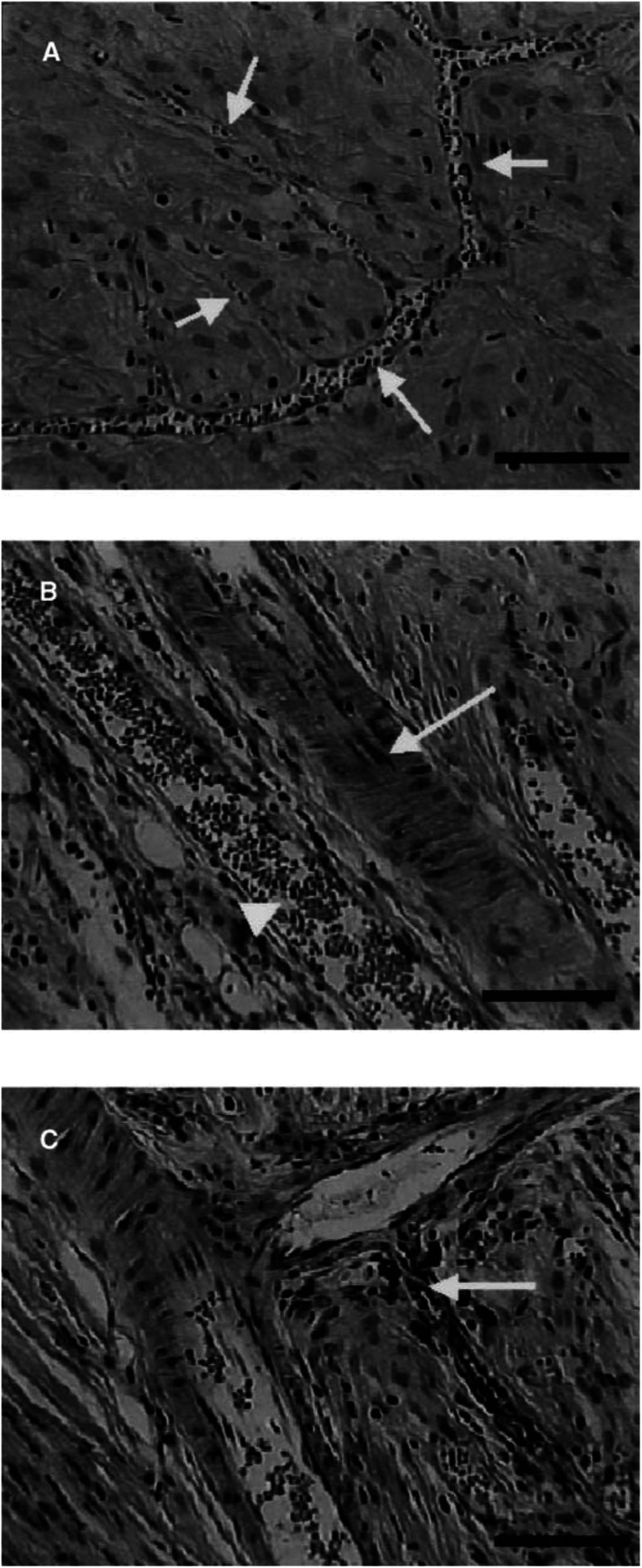
Normal microvessels after the administration of AC7700: (**A**) true capillaries (arrows); (**B**) arteriole (arrow) and venule (arrowhead); (**C**) arteriole and precapillary vessel (arrow). Bars, 100 *μ*m. Haematoxylin and eosin staining. A rat with a transparent chamber was killed 2 h after the administration of 10 mg kg^−1^ AC7700. The tissue was resected, and routine histological studies were performed. Note that the vascular lumens of the normal arterioles, venules, and even true capillaries (with diameters of 10–15 *μ*m) remained open after AC7700 administration.

In normal vessels, however, the blood flow did not stop after i.v. administration of 10 mg kg^−1^ AC7700. Even if the blood flow completely disappeared after death, the vascular lumen in the arterioles, true capillaries, and venules remained open (Figure 6

). Especially noteworthy is the fact that even in the true capillaries with diameters of 15 *μ*m or less, the vascular lumen did not close (Figure 6C).

### Effect of AC7700 on TIFP

#### Diffusion chamber method.

**Figure 7:**
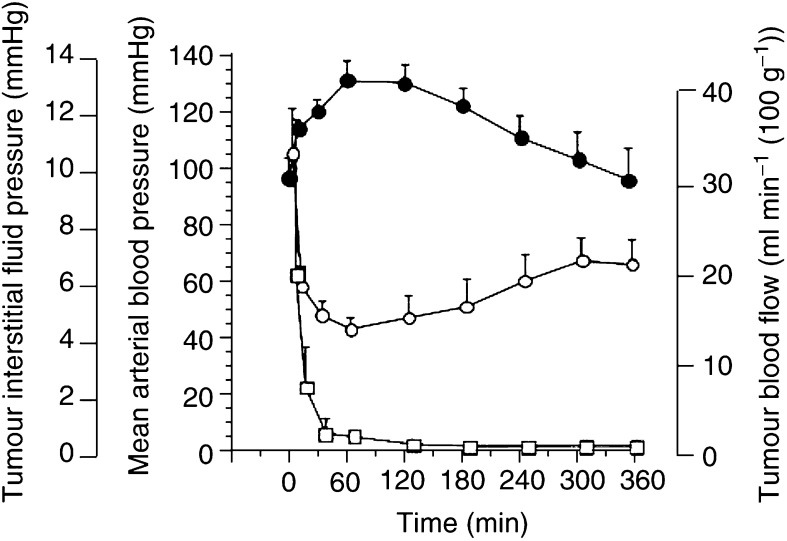
Simultaneous changes in TIFP, MABP, and TBF caused by AC7700. ○, TIFP; •, MABP; □, TBF. AC7700 administration (10 mg kg^−1^) was completed at 0 min. Note that TIFP markedly decreased, together with a TBF decrease, immediately after AC7700 administration.

When a diffusion chamber was implanted in the s.c. tissue of the back of a rat and a tumour cell suspension was injected around the chamber, the chamber was completely enveloped in the growing tumour 1 week later. Figure 7

shows the simultaneous changes in MABP, TIFP, and TBF (*n*=5) caused by 10 mg kg^−1^ AC7700. The mean tumour size, MABP, TIFP, and TBF at the start of measurement were 3.5±0.4 cm^3^, 96.4±7.1 mmHg, 10.5±1.6 mmHg, and 23.5±16.6 ml min^−1^(100 g)^−1^, respectively. When AC7700 was administered i.v., MABP increased, and TBF and TIFP simultaneously decreased. The MABP, TIFP, and TBF 30 min after AC7700 administration were 120.0±4.0 mmHg, 4.8±0.5 mmHg, and 2.5±1.7 ml min^−1^ (100 g)^−1^, respectively. These parameters did not change at all after i.v. administration of 0.9% NaCl solution (data not shown). Changes in MABP, TIFP, and TBF induced by AC7700 were all significant (MABP, *P*=0.0022; TIFP, *P*=0.0007; TBF, *P*=0.0388).

#### Wick-in-needle method.

At the start of measurement using the wick-in-needle method, the mean tumour size was 6.4±3.0 cm^3^ (*n*=6). After i.v. administration of 10 mg kg^−1^ AC7700, MABP significantly increased from 95.2±7.8 (before the drug) to 137.3±10.7 mmHg (30 min after administration) (*P*<0.0001), TIFP significantly decreased from 12.1±3.1 (before) to 7.1±2.3 mmHg (30 min after) (*P*=0.001), and TBF significantly decreased from 30.2±6.1 (before) to 5.5±1.9 ml min^−1^ (100 g)^−1^ (30 min after) (*P*<0.0001). The results obtained by this method supported those of the diffusion chamber method.

## DISCUSSION

We conclude that TBF stasis brought about by AC7700 administration most likely results from an indirect action involving the contractile response of arterioles, rather than a direct effect of this drug on tumour vessels. Our experiments in the present study strongly support this conclusion.

In the first experiment, the sensitivity of vessels in tumours to topical application of AC7700 was compared with that of vessels in normal s.c. tissue. Normal tissue was more sensitive than tumour tissue to this agent. When blood flow volume was measured via the hydrogen clearance method, what was actually measured was not the total blood flow (Gullino and Grantham, 1961), but the local blood flow (Peterson, 1979). The electrodes used in the experiment measured blood flow around the 1-mm tip of the electrode. After application of AC7700 to the region of measurement, notable changes in TBF were not found with concentrations up to 10 mg ml^−1^. In normal tissue, however, there was strong constriction of the arterioles with 1 mg ml^−1^ AC7700, as well as a large decrease in blood flow in the capillary bed. We therefore conclude that the small changes in TBF were not due to an insufficient volume of AC7700 having been applied to the region, but instead were due to the relatively low sensitivity of the tumour vessels themselves. Thus, it was strongly suggested that the stasis of TBF induced by AC7700 administration was not caused by a direct effect of the drug on the tumour vasculature.

In the second experiment, the effects of i.v. and i.a. administration of AC7700 were compared. If AC7700 works directly on tumour vessels, then the stasis effect after arterial administration – leading to a high concentration of AC7700 reaching the tumour – should be stronger than that after venous administration. In fact, stronger stasis was not obtained with i.a. administration. This finding strongly suggests that the site of AC7700 action is not the luminal surface of tumour vessels.

The results of the above experiments indicate that the primary action of AC7700 is not on the tumour vasculature. If that is the case, then why indeed does AC7700 bring about TBF stasis and why does not the stopped TBF subsequently recover? To answer these questions, we used a rat transparent chamber to observe the effects of AC7700 on host arterioles and the tumour vascular network.

We previously reported that the fact that the sites of increased vascular resistance differ with each vasopressor is the primary reason why various vasopressors produce different changes in TBF (Hori *et al*, 1993). Increases in arteriolar resistance upstream cause a decreases in the perfusion pressure of tumour-feeding vessels downstream. In the present study, AC7700 constricted a3 and a4 arterioles upstream, leading to increases in arteriolar resistance. In fact, TBF decreased continuously, blood flow stopped completely after 30 min of AC7700 administration, and the vessels themselves disappeared from sight. However, we also confirmed that when a small dose of AC7700 was used, the increased resistance of arterioles disappeared in 2–3 h, and the blood flow to the vessels feeding the tumour recovered (data not shown). We therefore conclude that the TBF stasis effect of AC7700 is triggered by continued constriction of host arterioles. It is not yet clear from the present studies alone as to why AC7700 constricts host arterioles. We consider that the receptor for AC7700 may be on the vascular smooth muscles. Further studies are necessary to demonstrate the hypothesis.

When blood flow in tumour-feeding vessels is stopped, that effect is immediately transmitted to the tumour capillaries. Before AC7700 administration, when TBF was strong, many vessels with diameters larger than 50 *μ*m were seen; subsequent to drug administration, these vessels had greatly constricted vascular lumens or completely disappeared. It is thought that this effect is caused by structural characteristics of tumour vessels, that is, tumour vessels are composed fundamentally of one layer of endothelial cells (Papadimitriou and Woods, 1975; Konerding *et al*, 1989), or that, even when many pericytes and smooth muscle cells are present around the tumour vessels, the binding between the tumour endothelial cells and pericytes or smooth muscle cells is weak (Morikawa *et al*, 2002). In general, the endothelium of tumour vessels is stretched, such that the vascular wall is extremely thin, and intravital microscopic observations have shown that lumen morphology is maintained with difficulty. The reason for the observation of many thread-like tumour vessels after AC7700 administration is that tumour vessels are fundamentally passive vessels, so that they dilate when blood flow increases and constrict when blood flow decreases. The constriction or disappearance of the lumen of tumour vessels clearly poses a significant resistance to reperfusion.

In contrast, in the case of normal capillaries, even when there is a decrease in blood flow the vascular lumen does not collapse, and the normal morphology is maintained. This finding was confirmed by observations of both intravital microscopic samples and tissue samples fixed in formalin. Such results are thought to be a consequence of both a strong binding between the endothelial cells and pericytes of normal capillaries and the strong structural support provided by a continuous basement membrane (Simionescu and Simionescu, 1984). Even when blood flow is temporarily blocked, reperfusion will not encounter a large resistance, provided that the vascular lumen is not destroyed.

The most dramatic change seen after AC7700 administration was in the drainage of the tumour vascular bed, which frequently has a sinusoid-like structure. In that region, blood flow is usually not rapid, and the administration of AC7700 led to further slowing and sluggishness. Ultimately, TBF stopped completely, and many erythrocytes stagnated there. After 2–3 h, those erythrocytes underwent haemolysis, resulting in thrombosis in the lumens of the drainage tumour vessels. It is well known that one of the causes of blood stasis is the formation of thrombus. Nilsson *et al* (2001) reported that tissue factor induces intratumour thrombus formation, resulting in the cessation of blood flow and widespread necrosis within the tumour. In the case of AC7700 administration, however, the first effect is blood flow cessation, followed by thrombus formation within the lumens of the drainage tumour vessels because of haemolysis.

Even after 2 h of occlusion of tumour-feeding vessels, recovery of blood flow is possible (data not shown), but if haemolysis occurs in the drainage region the TBF stasis becomes irreversible. Since haemolysis acts as a vascular toxin and is likely to be a cause of vascular destruction, it is thought to be the direct cause of irreversible TBF stasis. The precise reason for the haemolysis remains uncertain, and additional studies of this phenomenon are needed.

Recently, Tozer *et al* (2001, 2002) reported a mechanism of the stoppage of TBF brought about by CA-4-P, in which they emphasised the importance of the increase in TIFP. It was previously reported that TIFP increases together with tumour proliferation (Paskins-Hurlburt *et al*, 1982; Wiig *et al*, 1982; Hori *et al*, 1986; Jain, 1987). In some cases, it also increases after chemotherapy and radiotherapy (Roh *et al*, 1991; Curti *et al*, 1993). The increased TIFP due to increased tumour vascular permeability in turn is thought to compress tumour vessels and bring about decreases in TBF. In fact, Tozer *et al* (2001) demonstrated in *in vivo* experiments that CA-4-P increased tumour vascular permeability. Moreover, in the process of increasing permeability, CA-4-P bound to tubulin and was surmised to be involved in causing the cytoskeletal disorganisation of the endothelium (Dark *et al*, 1997; Grosios *et al*, 1999). Although these authors did not actually measure the TIFP, they argued that an increase in vascular permeability necessarily leads to an increase in TIFP. Eikesdal *et al* (2002), however, reported that in BT_4_An rat gliomas, CA-4-P did not produce an increase in TIFP. These conflicting findings suggest that there may be differences in tumour vascular permeability and TIFP brought about by CA-4-P that are related to the type of tumour.

Tozer *et al* (2001) also reported that there is no reduction in arteriolar or venular diameter at early times after i.v. administration of CA-4-P. In the case of AC7700, active constriction of arterioles was observed immediately after drug administration. Constriction of arterioles soon led to a decrease in blood flow in the tumour-feeding vessels. As there was a marked decrease in the volume of fluorescent dye reaching the tumour vessels as a result of the decrease in TBF (data not shown), we were unable to determine directly whether an increase in tumour vessel permeability followed AC7700 administration. However, the fact that there was an immediate, marked decrease in TIFP strongly suggests that AC7700 induced no increase in the permeability of tumour vessels. We surmise that AC7700 strongly constricts tumour-feeding vessels, and as a consequence of the marked suppression of the volume of water flowing into the tumour, a decrease in TIFP occurs.

The finding that CA-4-P does not immediately constrict the arteriolar system whereas AC7700 immediately constricts it suggests that their similar chemical structures result in similar effects via different responses in the vascular system. That the pharmacological effects of these two similar drugs differ, or that sometimes the drugs produce opposite effects, is not unusual in pharmacology. For example, it is well known that both epinephrine and isoproterenol are catecholamines with similar structures, but they produce diametrically opposed effects on peripheral vascular resistance. It would therefore not be surprising if AC7700 and CA-4-P also have different mechanisms of action.

**Figure 8:**
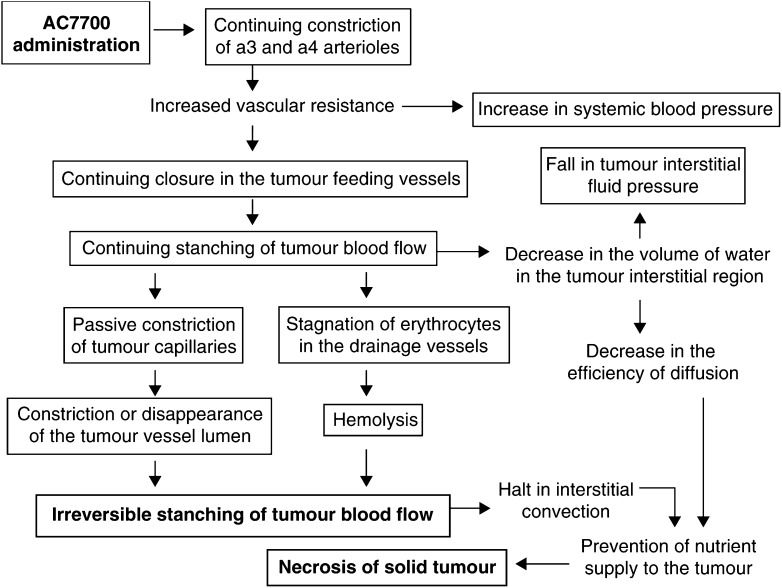
Microvascular mechanisms of AC7700-induced TBF stasis and necrosis formation. Initially, AC7700 causes the powerful and continuing constriction of pre-existing arteriolar vessels, which is why systemic blood pressure is raised after administration of the drug. With the continuing increase in the resistance of arterioles, a fall in perfusion pressure occurs in the tumour-feeding vessels downstream. As a result, blood flow to the tumour vascular bed halts, and that effect, in turn, causes a decrease in the volume of water in the tumour interstitial region, producing a decrease in TIFP. Owing to the complete cessation of TBF, the lumens of tumour vessels with their weak supporting structures become constricted or disappear entirely. The blood flow in the drainage vessels of the tumour vascular bed comes to a halt, leading to the accumulation of erythrocytes, and the erythrocytes undergo haemolysis in 2–3 h. Constriction or disappearance of the tumour vessel lumens and haemolysis cause the exit routes from the tumour to become closed, and recovery of TBF becomes impossible, which makes the stasis irreversible. The halt in TBF causes interstitial convection to stop, and the removal of water from the tumour interstices reduces the efficiency of diffusion. Decreases in both convection and diffusion prevent nutrient supply to the tumour, which is ultimately the cause of necrosis of the solid tumour tissue. Squares, observed or measured events.

In light of the experimental findings obtained in the present study, we can summarise the effects of AC7700 as producing TBF stasis that, in turn, brings about necrosis of solid tumours, as shown in Figure 8

. To obtain the widespread necrosis of tumour tissue as a result of TBF stasis, it is necessary and sufficient to close the entrance to the tumour vascular bed for more than 2 h and to occlude the exit routes via haemolysis. We have already demonstrated this effect by using drugs other than AC7700 and will report those results elsewhere. In the present study, we clarified the microcirculatory mechanism by which AC7700 brings about irreversible stasis of TBF, leading to tumour necrosis. In the future, it will be necessary to clarify at the molecular levels, using immunohistochemistry, whether there is an AC7700 receptor in the smooth muscle cells of blood vessels, and why haemolysis of erythrocytes trapped within tumour vessels occurs.
